# Targeting the Multidrug Transporter Ptch1 Potentiates Chemotherapy Efficiency

**DOI:** 10.3390/cells7080107

**Published:** 2018-08-14

**Authors:** Anida Hasanovic, Isabelle Mus-Veteau

**Affiliations:** 1 Université Côte d’Azur, Campus Valrose, 06100 Nice, France; hasanovic@ipmc.cnrs.fr; 2 CNRS UMR7275, Institut de Pharmacologie Moléculaire et Cellulaire, Sophia Antipolis, 06560 Valbonne, France; 3 NEOGENEX CNRS International Associated Laboratory, Sophia Antipolis, 06560 Valbonne, France

**Keywords:** Hedgehog receptor Ptch1, efflux pump, multidrug resistance, transporter, cancer therapy

## Abstract

One of the crucial challenges in the clinical management of cancer is resistance to chemotherapeutics. Multidrug resistance (MDR) has been intensively studied, and one of the most prominent mechanisms underlying MDR is overexpression of adenosine triphosphate (ATP)-binding cassette (ABC) transporters. Despite research efforts to develop compounds that inhibit the efflux activity of ABC transporters and thereby increase classical chemotherapy efficacy, to date, the Food and Drug Administration (FDA) has not approved the use of any ABC transporter inhibitors due to toxicity issues. Hedgehog signaling is aberrantly activated in many cancers, and has been shown to be involved in chemotherapy resistance. Recent studies showed that the Hedgehog receptor Ptch1, which is over-expressed in many recurrent and metastatic cancers, is a multidrug transporter and it contributes to the efflux of chemotherapeutic agents such as doxorubicin, and to chemotherapy resistance. Remarkably, Ptch1 uses the proton motive force to efflux drugs, in contrast to ABC transporters, which use ATP hydrolysis. Indeed, the “reversed pH gradient” that characterizes cancer cells, allows Ptch1 to function as an efflux pump specifically in cancer cells. This makes Ptch1 a particularly attractive therapeutic target for cancers expressing Ptch1, such as lung, breast, prostate, ovary, colon, brain, adrenocortical carcinoma, and melanoma. Screening of chemical libraries have identified several molecules that are able to enhance the cytotoxic effect of different chemotherapeutic agents by inhibiting Ptch1 drug efflux activity in different cancer cell lines that endogenously over-express Ptch1. In vivo proof of concept has been performed in mice where combining one of these compounds with doxorubicin prevented the development of xenografted adrenocortical carcinoma tumors more efficiently than doxorubicin alone, and without obvious undesirable side effects. Therefore, the use of a Ptch1 drug efflux inhibitor in combination with classical or targeted therapy could be a promising therapeutic option for Ptch1-expressing cancers.

## 1. Introduction

Despite the major progress that has been made in biomedical research, and the development of novel therapeutic strategies, cancer is still among the dominant causes of death worldwide [1]. One of the crucial challenges in the clinical management of cancer is primary (intrinsic) and secondary (acquired) resistance to both conventional and targeted chemotherapeutics.

Cancer is a complex disease and it represents the result of the progressive accumulation of genetic aberrations and epigenetic changes that escaped from the regular cellular and environmental controls. Cancer cells usually acquire genetic aberration including aneuploidy, chromosomal rearrangement, loss or gain of function mutations, deletions, gene rearrangements and amplifications [2].

Chemotherapy is one of the major treatments for cancer. It was first discovered with the application of alkylating agents in the 1940s [3,4,5]. Although chemotherapy has led to an improvement in survival and life quality for cancer patients, the majority of them develop progressive disease after an initial response to the treatment. This chemotherapy resistance is a major issue in the clinical management of cancer. There are two different types of resistance to chemotherapy: (1) acquired resistance that develops after exposure to the drugs, and (2) intrinsic resistance which is present even before the exposure to the chemotherapeutic agents [6].

Multidrug efflux is one of the most important chemotherapy resistance mechanisms. The adenosine-triphosphate-binding-cassette (ABC) transporter superfamily transports toxins, sugars, amino acids, nucleotides, and metabolites out of cells [5], and protects cells from all living species against toxic molecules, including drugs. These transporters also contribute to a decrease in the cellular accumulation of hydrophobic anticancer drugs by expelling them from cells (Table 1).

ABC transporters have a conserved structure, with nucleotide binding domains (NBD) and transmembrane domains (TMD) [7]. The drug transport mechanism of ABC transporters is coupled to ATP hydrolysis [8]. There are 49 ABC transporters classified in seven subfamilies, which are expressed in both normal and malignant cells [9,10]; the transporters that are most frequently involved in multidrug resistance (MDR) are P-glycoproteins (P-gp; MDR1/ABCB1), MDR-associated proteins (MRP1/ABCC1) and breast cancer resistance proteins (BCRP/ABCG2).

Since the discovery that the over-expression of ABC transporters in cancer cells can mediate resistance to anti-cancer drugs, research has been directed towards developing compounds that inhibit the efflux activity of these transporters, and thereby increase classical chemotherapy efficacy. However, P-gp is expressed in normal tissues such as at the apical surface of liver hepatocytes, in the proximal tubular cells of kidneys, and in enterocytes of the intestines, where it has a physiological function in the detoxification of the organism by excreting its substrates into bile, urine, and the intestinal contents, which explains why the use of P-gp inhibitors leads to undesirable consequences. ABC transporters were also found to be expressed in other tissues such as the heart, where they have a role in detoxification and protection of the heart from the accumulation of xenobiotics. Many cases of cardiotoxicity have been linked to an increase in drug concentrations in the heart after co-administration of antineoplastic drugs and multidrug-resistance-reversing agents (drugs that are identified as P-gp inhibitors, and that have the capability to restore the drug sensitivity of antineoplastic-resistant tumor cells) [11]. Moreover, the antidiarrheal loperamide has been shown to enter into the central nervous system (CNS) (from which it is normally excluded) by the simultaneous administration of the P-gp modulator quinidine, and genetic disruption of P-gp in mice enhances levels of substrate drugs accumulated in the brain, with markedly slower elimination from the circulation, resulting in dramatically increased toxicity to normal tissue [12]. Thus, the simultaneous use of cytotoxic drugs and agents that block P-gp function has raised questions of safety. Therefore, to date, the Food and Drug Administration (FDA) has not approved the use of any ABC transporter inhibitors due to toxicity issues [13]. 

It has been reported that the Hedgehog (Hh) signaling pathway, which is aberrantly activated in many cancers, upregulates the expression of certain ABC transporters such as P-gp and BCRP [14,15]. The Hh signaling pathway controls cell differentiation and proliferation, and also plays a crucial role in embryonic development, and in stem cell homeostasis and tissue regeneration in the adult [16,17]. In vertebrates, canonical Hh signaling can be initiated by three ligands: Desert hedgehog (Dhh), Indian hedgehog (Ihh), and Sonic hedgehog (Shh). Shh is the most frequently studied Hh ligand. It is expressed in the central nervous system, lungs, teeth, intestines, and hair follicles during development. Later, during organogenesis, Shh is expressed in, and affects development of most epithelial tissues. Ihh is involved in endochondral bone formation, as a negative regulator of chondrocyte differentiation, and it participates in the development of gastrointestinal tract and mammary glands. Dhh is the closest homologue of the *Drosophila* Hh ligand. Its expression is restricted to the gonads, including the Sertoli cells in the testis, where it plays a key role in the differentiation of germ cells. All Hh proteins undergo maturation before the active ligand is released from the cell and activates Hh signaling. The 45 kDa long polypeptide is auto-catalytically cleaved to produce the active Hh protein, a ~19 kDa N-terminal Hh signaling domain (HhN) with cholesterol at the C-terminus and a palmitic acid moiety at the N-terminus. The secretion of mature, functionally active Hh proteins is regulated by Dispatched (Disp) protein, which allows their release from the cells. Hh proteins can act as mitogens, morphogens, and differentiation factors at longer or shorter distances, during different stages of development and in different tissues. Shh, Ihh, and Dhh ligands bind with similar affinity to both of the two Patched homologs isolated in vertebrates, which both repress the activity of Smo protein in the absence of ligand. Ptch1 is primarily expressed in mesenchymal cells that produce Shh proteins, while Ptch2 is expressed in skin and testicular epithelial cells [17]. Binding of Hh ligand to Ptch1 leads to its internalization and degradation. This process relieves the inhibitory effect of Ptch1 on Smo, which then activates the Gli zinc-finger transcription factors; these factors control the transcription of Hh target genes, including Fox, Myc, Patched, Hhip, Snail, Nanog, Sox2, and cyclin D, which are involved in cell development, differentiation, epithelial-mesenchymal transition (EMT), and stem cell maintenance [18,19,20]. Aberrant activation of Hh signaling has been shown to be involved in the initiation, promotion, metastasis, and chemotherapy resistance of a growing number of solid and hematologic tumors [21,22]. This is the case in particular for cells that exhibit resistance to chemotherapy, such as cancer stem cells or tumor-initiating cells [20].

## 2. The Hh Receptor Ptch1 Is Overexpressed in Many Aggressive Cancers

The major mechanisms by which the Hh pathway is aberrantly activated in cancer can be attributed to mutations of Hh pathway constituents (Type I: ligand-independent), excessive expression of Hh pathway ligands (Type II–IIIb: ligand-dependent), and the generation of a cancer stem cell (CSC) phenotype (Type IV) (Table 2) [20].

The link between Hh signaling and tumorigenesis was first discovered in patients with Gorlin syndrome, which was characterized by a loss of Ptch1 heterozygosity [23,24,25,26]. Gorlin syndrome also known as nevoid basal cell carcinoma syndrome (NBCCS), is an autosomal dominant disorder that predisposes patients to medulloblastomas, basal cell carcinomas (BCCs) and developmental defects [23]. NBCCS patients are also at an increased risk for ovarian fibromas, meningiomas, fibrosarcomas, rhabdomyosarcomas, cardiac fibromas, and ovarian dermoids. These are type I—ligand-independent cancers with autonomous Hh signaling. Other mechanisms of Hh signaling involved in cancer development are ligand-dependent. For example, in some cancers, tumor cells produce Hh, which then activates the Hh signaling in themselves in an autocrine manner (Type II—ligand-dependent signaling in autocrine manner) [20]. This type of activation was based on tumors that express both the Hh ligand and the downstream Hh components. Other tumors function through paracrine effects on the surrounding stroma (Type IIIa—Ligand-dependent, paracrine signaling) [27,28]. For example, pancreatic, ovarian, prostate, and colorectal cancers activate Hh signaling via paracrine stimulation [29,30]. In type IIIb—ligand-dependent, reverse paracrine signaling cancers, stromal cells produce and secrete Hh ligand which then activate Hh signaling in tumor cells [27,31]. This is the case in hematological malignancies such as B-cell lymphoma, multiple-myeloma and leukemia, in which Hh secreted from the bone marrow stroma is essential for the survival of cancerous B cells through the upregulation of the anti-apoptotic factor Bcl-2 [32,33]. In a fourth type of cancer, Hh signaling is involved in the maintenance of a subpopulation of tumor cells that exhibit stem cell-like properties. This rare subset of tumor-initiating cells, termed cancer stem cells (CSCs), are proposed to maintain a self-renewing reservoir, and they differentiate into transient amplifying cells to produce a state of cellular heterogeneity within a tumor [34]. Hh signaling is believed to drive the CSC phenotype through the regulation of stemness-determining genes such as *Nanog*, *Oct4*, *Sox2* and *Bmi1*. While Hh-driven CSCs have been validated for numerous hematological malignancies, their existence in solid tumors remains more controversial [18].

Mounting evidence indicates that ligand-independent Hh signaling, also called non-canonical Hh signaling plays an essential role in cancer. In some situations, the Gli transcription factors can be activated by other molecules/signaling independent of ligand and Smo. The molecules/signaling pathways that can bypass the ligand-receptor signaling axis to activate Hh signaling, including Kras signaling, TGFβ, PI3K, PKC, and epigenetic regulators [35]. Moreover, a vast number of studies have demonstrated that not all non-canonical Hh signaling proceeds through Gli activation. There are two classes of Gli-independent, non-canonical Hh signaling in cancers: type I, which works through Ptch1 and is independent of Smo, and type II, which functions through Smo activation [36]. 

**Figure 1:**
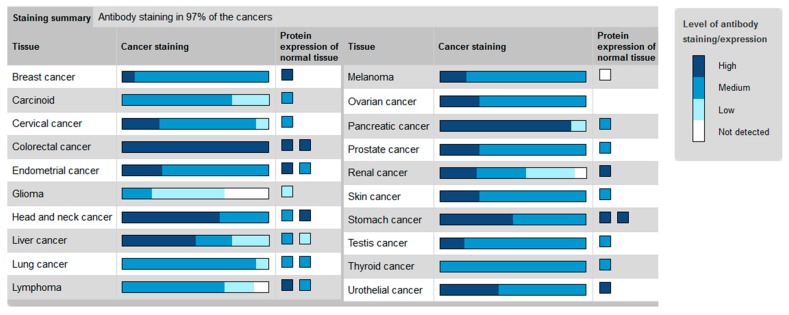
Ptch1 protein level in cancers. From the Protein Atlas website http://www.proteinatlas.org/ENSG00000185920-PTCH1/cancer [40].

As the Hh receptor Ptch1 is an Hh target gene, this receptor has been shown to be overexpressed in many cancers such as lung, breast, prostate, ovary, colon, brain, melanoma [21,37,38], and myeloid leukemia [15,39] (see the Human Protein Atlas website http://www.proteinatlas.org/ENSG00000185920-PTCH1/cancer, [40]) (Figure 1).

Indeed, in 2013, a study from Korea examined 334 cases of breast cancer and found that Ptch1 was overexpressed in 190 of them, and was significantly correlated with lymph node metastasis, advanced cancer stages, and more aggressive tumor behavior [41]. This was confirmed in a recent study that was performed on 150 fresh tumors from breast cancer patients, which reported that Ptch1 was significantly overexpressed in tumors, as compared to in corresponding normal mammary tissues [42]. According to a review article from Papadopoulos and co-workers [43], Ptch1 is also upregulated in colorectal cancers. Further, immunohistochemical (IHC) analysis on adrenocortical carcinoma (ACC) samples from 70 patients showed that Ptch1 was expressed in ACC tumor tissues from all 70 patients [44]. Finally, Ptch1 has been proposed to be an early marker for gastric and thyroid cancers [45,46].

A study published in 2010 [47] reported that high levels of Ptch1 were detected in 76% of biopsy specimens from esophageal squamous cell carcinoma patients that were treated with chemotherapy. Interestingly, significant associations were observed between high Ptch1 and Gli1 expression with large tumor size, locoregional progression, and an incomplete response to chemotherapy. In a multivariate analysis, Ptch1 and Gli1 expression status were both evaluated as independent prognostic factors for locoregional progression-free survival, distant progression-free survival, and overall survival; the authors observed that Ptch1 and Gli1 expression may be significantly associated with resistance to chemotherapy in esophageal squamous cells carcinoma. This is strengthened by a very recent study reporting that Ptch1 expression correlates with, and may be a prognostic marker for biochemical relapse in high-risk prostate cancer patients [48]. 

Hence, there are multiple lines of evidence that suggest that Ptch1 expression may contribute to cancer resistance to chemotherapy. The question is, how?

## 3. Ptch1 Is a Multidrug Transporter Involved in Chemotherapy Resistance

The regulation of Smo activation by Ptch1 is altered in many cancers. The mechanism by which Ptch1 represses Smo has been the subject of numerous studies. For example, Taipale and co-workers [49] showed in 2002 that Ptch1 inhibits Smo sub-stoichiometrically, suggesting that there is no direct interaction between Ptch1 and Smo. Several small molecules modulate Hh signaling through direct binding to Smo, and some Smo antagonists are in clinical trials for treating tumors [21,50]. Moreover, several studies have reported that Smo can be repressed by molecules such as vitamin D_3_ [51], and activated by oxysterols [52,53,54] and phosphatidylinositol 4-phosphate [55]. On the basis of these observations and the sequence homology of Ptch1 with bacterial transporters, Ptch1 has been proposed to function as a transporter that could change the concentration of small molecules involved in Smo activation or inhibition [49,56].

**Figure 2:**
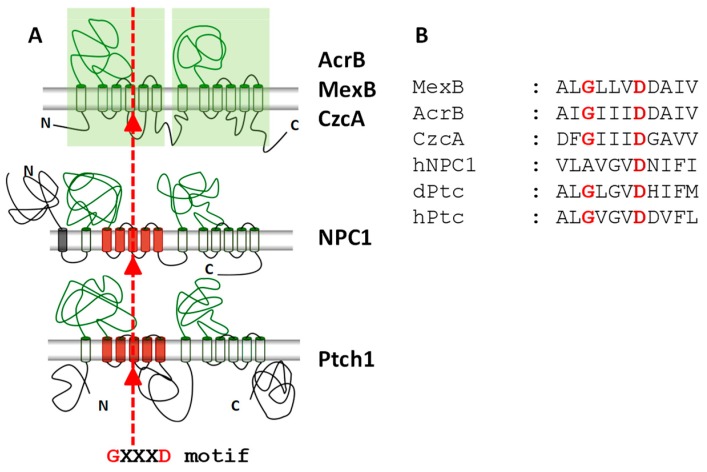
Ptch1 sequence homologies with the Niemann-Pick disease type C1 protein (NPC1) and the bacterial transporters from the RND family such as AcrB, MexB and CzcA. (**A**) Protein topology. The sterol sensing domains (SSD) are represented in red, and the transmembrane segment containing the highly conserved GXXXD motif is indicated. (**B**) Sequence alignment of the highly conserved GXXXD motif.

Secondary structure prediction suggests that Ptch1 has 12 transmembrane-spanning domains, two hydrophilic extracellular loops, and intracellular N- and C-terminal domains (Figure 2). 

**Figure 3:**
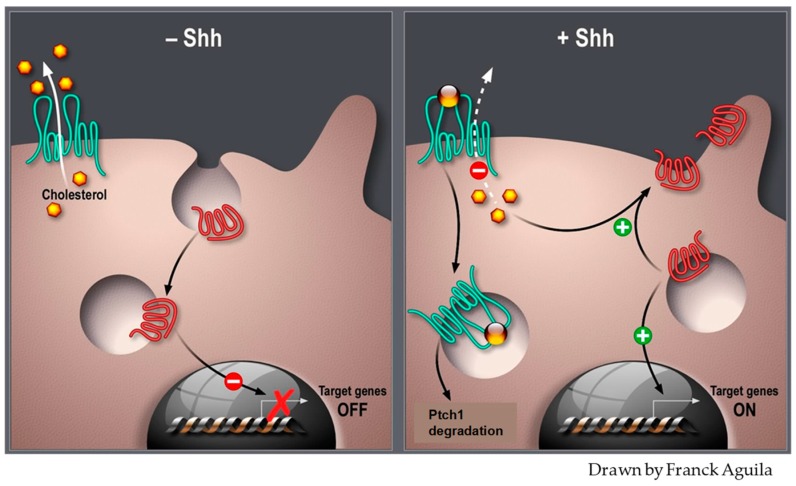
Ptch1 cholesterol transport activity regulates Hedgehog signaling. Ptch1 is represented in green, Smoothened in red, cholesterol in yellow, and Shh as a golden ball.

In view of the sequence similarity between Ptch1 and the Niemann-Pick disease type C1 protein (NPC1) which is involved in cholesterol binding and transport [57,58,59,60], Bidet and co-workers investigated the relationship between Ptch1 and cholesterol, and demonstrated that Ptch1 is also involved in cholesterol binding and transport [61]. The Ptch1 sequence possesses a sterol-sensing domain (SSD), which is a phylogenetically conserved domain that is shared by several classes of proteins with key roles in different aspects of cholesterol homeostasis, such as NPC1 [62] (Figure 2). This domain is essential for Smo repression in *Drosophila* and vertebrates [63,64]. Indeed, blocking Ptch1 SSD activity in *Drosophila* causes not only endosomal lipid accumulation, but also alters the trafficking of Smo from endosomes [65]. Using the well-known Sonic Hedgehog (Shh)-responding mouse fibroblast cell line NIH3T3, Bidet and colleagues [61] observed that enhancing the intracellular cholesterol concentration induced Smo enrichment in the plasma membrane, which is a crucial step for signaling activation. They found that binding of Shh protein to its receptor Ptch1, which involves Ptch1 internalization, increased the intracellular concentration of cholesterol and decreased the efflux of a fluorescent cholesterol derivative (BODIPY-cholesterol) in these cells. They observed that treatment of fibroblasts with cyclopamine, an antagonist of Hh signaling, inhibited Ptch1 expression and reduced BODIPY-cholesterol efflux, while treatment with the Smo agonist SAG enhanced Ptch1 protein expression and BODIPY-cholesterol efflux. They also showed that over-expression of human Ptch1 in the yeast *S. cerevisiae* resulted in a significant boost of BODIPY-cholesterol efflux. Finally, they demonstrated that purified Ptch1 is able to bind to cholesterol, and that the interaction between Shh and Ptch1 inhibits the binding of Ptch1 to cholesterol. These results demonstrated for the first time that Ptch1 contributed to cholesterol efflux from cells. This activity is likely to be responsible for the inhibition of Smo enrichment at the plasma membrane, which is a crucial step in Hh pathway activation (Figure 3). The involvement of Ptch1 in cholesterol transport and the Ptch1-dependent Smo regulation mechanisms proposed in this study are consistent with the impairment of Hh signaling caused by decreased intracellular cholesterol levels that is observed in congenital malformations such as Smith-Lemli-Opitz syndrome (SLOS), desmosterolosis, and lathosterolosis [56]. Defective regulation of cholesterol biosynthesis could further aggravate impaired Hh signaling in holoprosencephaly which is the most severe form of SLOS [66]. This is also consistent with recent data suggesting that the binding of Hh to Ptch1 de-represses the levels of phosphatidylinositol 4-phosphate (PI4P), which in turn promotes Smo activation [55], since cholesterol has been shown to modulate PI4P synthesis [67].

Interestingly, Ptch1 possesses a GXXXD motif in the middle of TM4. This motif is highly conserved in the resistance-nodulation-division (RND) family of prokaryotic efflux pumps (Figure 2), and has shown to be associated with the transport activity of these proteins. Notably, of the many mutations in Ptch1 that are known to be associated with Gorlin’s syndrome [68], three affect these two conserved residues, strongly suggesting the existence of a function that is conserved between the RND family and Ptch1. These mutations were shown to inhibit Ptch1 suppression activity on the Hh pathway [49]. Moreover, Soloview and co-workers reported in 2011 that one of the two highly related Ptch1 gene homologs in the nematode *C. elegans*, PTC-3, has retained essential roles in *C. elegans* that are independent of Smo [69]. Missense changes in the transporter GXXXD/E motif revealed that the transporter domain is essential for PTC-3 activity, and suggested that the movement of lipids or sterols could represent a fundamental and evolutionarily conserved function for these proteins. 

Transporters from the RND family use the proton motive force to efflux a wide variety of substrates such as sterols, lipids, bile salts, fatty acids, and metal ions, as well as lipophilic drugs from the cytosol of Gram-negative bacteria [70,71], and are involved in drug resistance. In a study published in 2012, Bidet and colleagues showed for the first time that Ptch1 is a multidrug transporter involved in the resistance of cancers to chemotherapy [72]. They reported that the expression of Ptch1 in *S. cerevisiae* increased the efflux of doxorubicin, and conferred resistance on yeast to this chemotherapeutic agent, which is used to treat recurrent cases of cancers. They showed that this doxorubicin efflux was dependent on the proton motive force, and that yeast expressing Ptch1 protein carrying the double mutation G509VD513Y in the transport GXXXD motif were significantly less resistant to doxorubicin, and transported significantly less doxorubicin than yeast expressing wild-type Ptch1. The authors also observed that the expression of Ptch1 in yeast confers resistance to other molecules used in chemotherapy such as methotrexate, temozolomide, and 5-fluorouracil, to antibiotics such as hygromycin B, to antiseptics such as acriflavine, and to dyes such as H33342. They also showed that Ptch1 expression allows yeast to efflux the antiseptic acriflavine as efficiently as doxorubicin. To determine if Ptch1 also plays a role in drug efflux when endogenously expressed in mammalian cells, they studied doxorubicin efflux in the highly Shh-responsive mouse fibroblasts NIH3T3. They observed that the decrease in Ptch1 protein levels induced by the addition of Shh ligand during efflux measurements, or pre-treatment of fibroblasts with the Smo antagonist cyclopamine, significantly decreased the doxorubicin efflux from fibroblasts. Decreased Ptch1 protein levels also increased doxorubicin accumulation in melanoma and leukemia cell lines known to aberrantly express Hh signaling components [15,73]. Furthermore, they showed that the presence of Shh increased the cytotoxicity of doxorubicin on melanoma cells. 

The hypothesis that Ptch1 could be a multidrug transporter involved in chemotherapy resistance has been strongly strengthened by the observation, using Ptch1-silencing RNA, that doxorubicin efflux from the human cell line H295R, which was isolated from a patient diagnosed with an adrenocortical carcinoma (ACC), mainly occurs through Ptch1 and not through ABC transporters such as P-gp [44]. Notably, doxorubicin is one of the three chemotherapeutic agents that is included in the gold standard ACC therapy which often fails due to resistance to the treatment, leading to poor overall patient survival [74,75].

## 4. Patched Drug Efflux Activity and Cancer Cell Metabolism

Normal cells primarily produce energy through mitochondrial oxidative phosphorylation. The metabolism of glucose, the central macronutrient, allows for energy to be harnessed in the form of ATP, through the oxidation of its carbon bonds. In mammals, the end product can be lactate or, upon full oxidation of glucose via respiration in the mitochondria, CO_2_. This process is essential for sustaining all mammalian life. 

In tumors and other proliferating or developing cells, the rate of glucose uptake dramatically increases and lactate is produced, even in the presence of oxygen and in fully functioning mitochondria. This is called aerobic glycolysis, also termed the Warburg effect [76]. Aerobic glycolysis is less efficient than oxidative phosphorylation in terms of ATP production, but leads to the increased generation of additional metabolites that may particularly benefit proliferating cells. Increased glycolysis leads to increased glucose consumption and the production of lactate. The “Warburg effect” implies that cancer cells efflux lactate, which decreases the extracellular pH. The resulting pattern of an acidic extracellular environment and an alkaline cytosol is considered to be a hallmark of malignant cancers, and is referred to as a “reversed pH gradient” [77]. High lactate levels indicate metastasis, tumor recurrence, and low survival prognosis in some patients [78]. Accordingly, in 1996, Gerweck and Seetharama reported that electrode-measured pH values of tumors and adjacent normal tissues, which were concurrently measured by the same investigator in the same patients, consistently showed that the electrode measured pH (believed to primarily represent tissue extracellular pH) was substantially and consistently lower in tumor tissue than in normal tissue. Consistent with this, studies based on ^31^P-magnetic resonance spectroscopy have estimated that intra-cellular pH is essentially identical to or slightly more basic in tumors than in normal tissue [79]. 

Bidet and co-workers showed that Ptch1 uses the proton gradient to efflux chemotherapeutic agents [72]. This is possible only in tumor and in proliferating or developing cells, which present intracellular pHs that are higher than the extracellular pHs, due to the Warburg effect. Ptch1 drug efflux activity is then specific to cancer cells, in contrast to ABC transporters efflux activity, which also occurs in normal tissues. Ptch1 drug efflux inhibitors potentially represent much more cancer-specific and less toxic therapeutics than ABC transporters antagonists. This makes Ptch1 a particularly attractive therapeutic target to enhance the effectiveness of classical chemotherapeutic treatments, and to decrease the risk of recurrence and metastasis in cancers expressing Ptch1. 

## 5. Inhibition of Ptch1 Drug Efflux Activity Increases Chemotherapy Efficacy

A screening assay was developed to identify molecules that are able to inhibit the drug efflux activity of Ptch1 [80]. This assay used Ptch1-expressing-yeast that were able to grow in the presence of a chemotherapeutic agent, such as doxorubicin [72]. Several molecules that were capable of inhibiting the growth of Ptch1-expressing-yeast in the presence of doxorubicin were identified. One of these molecules is a natural compound purified from a marine sponge. This molecule (panicein A hydroquinone) was shown to inhibit doxorubicin efflux and to increase doxorubicin cytotoxicity in vitro, in human melanoma cells that endogenously over-express Ptch1. This compound was the first inhibitor of Ptch1 doxorubicin efflux activity to be described [81]. 

The screening of a collection of 1200 small drug and drug-like molecules allowed the discovery of a second inhibitor of Ptch1 drug efflux activity. In a recent study, Hasanovic and co-workers [44] showed that methiothepin, a serotonin transporter antagonist [82,83], significantly enhances the cytotoxic, pro-apoptotic, anti-proliferative, and anti-clonogenic effects of doxorubicin on ACC cells by inhibiting the doxorubicin efflux activity of Ptch1. They observed the same effect of methiothepin on cells that had been rendered resistant to doxorubicin. Interestingly, these doxorubicin-resistant cells showed an increased level of Ptch1 expression, suggesting that their resistance to the treatment was due to Ptch1 upregulation. These results demonstrated that methiothepin strongly enhances the efficacy of doxorubicin on ACC cells expressing Ptch1. In vivo experiments performed on mice bearing human ACC cells xenografts showed that the addition of methiothepin to doxorubicin treatment inhibited tumor growth more significantly than doxorubicin alone, in particular by enhancing doxorubicin accumulation in tumors. Notably, these effects were achieved without obvious undesirable side effects, and were specific, without increasing amounts of doxorubicin levels in the heart tissues of treated animals. This is a strong advantage in comparison to ABC transporter antagonists. Indeed, it was found that the co-administration of doxorubicin and the P-gp antagonist, verapamil, increased the concentration of doxorubicin in the heart of mice by 40%. The co-administration augmented the incidence and severity of degenerative changes in cardiac tissue, and decreased the survival rate compared with doxorubicin alone [84]. Other studies in rodents demonstrated that two other P-gp antagonists, cyclosporine A or its analog, PSC833, could also increase doxorubicin concentrations in correlation with a greater incidence and severity of myocardial damage [85,86]. The mechanism involved is probably related to an accumulation of drugs in the heart as a result of inhibition of the normal protective function of P-gp, or other ABC transporters, by multidrug-resistance-reversing agents.

Moreover, experiments showed that methiothepin is also able to inhibit doxorubicin efflux and to increase doxorubicin cytotoxicity on colorectal, breast, and melanoma cancer cells that endogenously over-express Ptch1.

**Figure 4:**
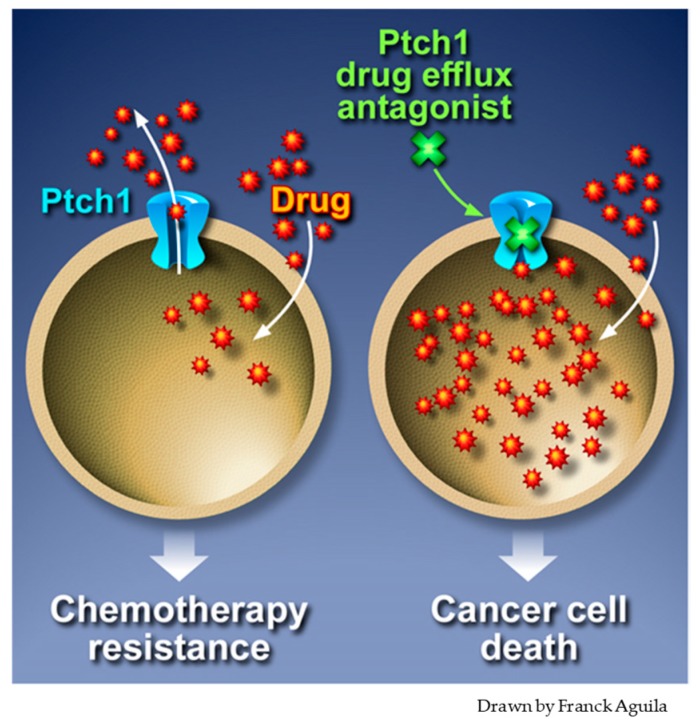
Combination of Ptch1 drug efflux inhibitor and chemotherapy is a potential new therapeutic option to overcome the drug resistance of cancer cells. Ptch1 inhibitor (in green) inhibits the doxorubicin (in red) efflux activity of Ptch1 (in blue). This allows the concentrations of doxorubicin to be reached that are required to kill cancer cells and fight chemotherapy resistance.

These results suggest that Ptch1 drug efflux inhibition could improve the effectiveness of doxorubicin on Ptch1-expressing cancers (Figure 4). Doxorubicin has been in use for over five decades as the backbone of chemotherapy treatment regimens for a wide range of cancers. However, due to risk of heart failure, the maximum life time cumulative dose of doxorubicin has been limited, decreasing the benefits that patients may receive from this potent drug [87]. Since methiothepin strongly increases doxorubicin efficacy and specifically inhibits Ptch1 doxorubicin efflux in cancer cells, the effective dose of doxorubicin received by patients could be reduced, thereby inducing less impairment in cardiac function.

## 6. Conclusions

It has long been postulated that the multidrug efflux transporter P-glycoprotein (P-gp/ABCB1/MDR1) mediates the main mechanism of resistance of cancer cells to chemotherapeutic agents; however, recent studies have shown that the Hh receptor Ptch1, which is over-expressed in many recurrent and metastatic cancers, pumps chemotherapeutic agents such as doxorubicin out of cancer cells, and thereby also contributes to chemotherapy resistance. Therefore, the use of an inhibitor of Ptch1 drug efflux activity in combination with classical or targeted chemotherapy could be a promising therapeutic option for Ptch1-expressing cancers.

## Figures and Tables

**Table 1:** Chemotherapy substrates for ATP-binding cassette (ABC) transporters (adapted from [5]).

Name	Exogenous Chemotherapy Substance
MDR1, ABCB1, P-GP	Anthracyclines (doxorubucin, daunorubicin, epirubicin), actinomycin D, colchicine, podophyllotoxin (etoposide, teniposide), methotrexate (only in carrier-deficient cells), mitomycin C, mitoxantrone, taxenes (paclitaxel, docetaxel), vinca alkaloids (vincristine, vinblastine)
MRP1, ABCC1	Anthracyclines, cochicine, etoposide, heavy metals (arsenite, arsenate, antimonials), vincristine, vinblastine, paclitaxel
MRP2, ABCC2, cMOAT	Cisplatin, CPT-11, doxorubicin, etoposide, methotrexate, SN-38, vincristine, vinblastine
MRP3, ABCC3	Cisplatin, doxorubicin, etoposide, methotrexate, teniopside, vincristine
MRP4, ABCC4	Methotrexate, nucleotide analogs, PMEA *
MRP5, ABCC5	Doxorubicin, methotrexate, nucleotide analogs, topotecan
MRP6, ABCC6	Doxorubicin, etoposide, teniposide
MRP8, ABCC11	5′-Fluorouracil, 5′-fluoro-2′-deoxyuridine, 5′-fluoro-5′-deoxyuridine, PMEA*
BCRP, ABCG2, MXR1, ABCP	Anthracyclines, bisantrene, camptothecin, epirubicin, flavopiridol, mitoxantrone, S-38, topotecan

* PMEA: 2′,3′-dideoxycytidine 9′-(2′-hosphonylmethoxynyl)adenine.

**Table 2:** Modes of signaling in Hedgehog (Hh) pathway-dependent cancer.

	Hh Signaling	Example of Cancers
Type I	Mutations on Ptch1, Smo, or suppressor of Fused (SUFU). Ligand independent cancers with autonomous Hh signaling	Nevoid basal cell carcinoma syndrome (NBCCS), medulloblastomas, basal cell carcinomas (BCCs), rhabdomyosarcoma
Type II	Ligand dependent with autocrine activation	Small-cell lung cancer, prostate, pancreatic, breast cancers
Type IIIa	Ligand-dependent, paracrine activation	Pancreatic, ovarian, prostate and colorectal cancers
Type IIIb	Ligand-dependent, reverse paracrine activation	B-cell lymphoma, multiple-myeloma and leukemia
Type IV	Regulation of stemness-determining genes	Cancer stem cells present in hematological malignancies and in solid tumors

## References

[1] Du C., Fang M., Li Y., Li L., Wang X. (2000). Smac, a mitochondrial protein that promotes cytochrome c-dependent caspase activation by eliminating IAP inhibition. Cell.

[2] Fidler I.J. (2001). Regulation of neoplastic angiogenesis. J. Natl. Cancer Inst. Monogr..

[3] Jacobson L.O., Spurr C.L., Guzman E.S., Barron E.S., Smith T., Lushbaugh C., Dick G.F. (1946). Nitrogen mustard therapy. Studies on the Effect of Methyl-Bis (Beta-Chloroethyl) Amine Hydrochloride on Neoplastic Diseases and Allied Disorders of the Hemopoietic System. JAMA.

[4] Goodman L.S., Wintrobe M.M. (1946). Nitrogen mustard therapy; use of methyl-bis (beta-chloroethyl) amine hydrochloride and tris (beta-chloroethyl) amine hydrochloride for Hodgkin’s disease, lymphosarcoma, leukemia and certain allied and miscellaneous disorders. J. Am. Med. Assoc..

[5] Choi C.H. (2005). ABC transporters as multidrug resistance mechanisms and the development of chemosensitizers for their reversal. Cancer Cell Int..

[6] Prieto-Vila M., Takahashi R.U., Usuba W., Kohama I., Ochiya T. (2017). Drug Resistance Driven by Cancer Stem Cells and Their Niche. Int. J. Mol. Sci..

[7] Amin L. (2013). P-glycoprotein Inhibition for Optimal Drug Delivery. Drug Target Insights.

[8] Ambudkar S.V., Kimchi-Sarfaty C., Sauna Z.E., Gottesman M.M. (2003). P-glycoprotein: From genomics to mechanism. Oncogene.

[9] Ozben T. (2006). Mechanisms and strategies to overcome multiple drug resistance in cancer. FEBS Lett..

[10] Stavrovskaya A.A., Stromskaya T.P. (2008). Transport proteins of the ABC family and multidrug resistance of tumor cells. Biochemistry.

[11] Couture L., Nash J.A., Turgeon J. (2006). The ATP-binding cassette transporters and their implication in drug disposition: A special look at the heart. Pharmacol. Rev..

[12] Luqmani Y.A. (2005). Mechanisms of drug resistance in cancer chemotherapy. Med. Princ. Pract..

[13] Jaramillo A.C., Al Saig F., Cloos J., Jansen G., Peters J.G. (2018). How to overcome ATP-binding cassette drug efflux transporter-mediated drug resistance?. Cancer Drug Resist..

[14] Sims-Mourtada J., Izzo J.G., Ajani J., Chao K.S. (2007). Sonic Hedgehog promotes multiple drug resistance by regulation of drug transport. Oncogene.

[15] Queiroz K.C., Ruela-de-Sousa R.R., Fuhler G.M., Aberson H.L., Ferreira C.V., Peppelenbosch M.P., Spek C.A. (2010). Hedgehog signaling maintains chemoresistance in myeloid leukemic cells. Oncogene.

[16] Varjosalo M., Taipale J. (2008). Hedgehog: Functions and mechanisms. Genes Dev..

[17] Skoda A.M., Simovic D., Karin V., Kardum V., Vranic S., Serman L. (2018). The role of the Hedgehog signaling pathway in cancer: A comprehensive review. Bosn. J. Basic Med. Sci..

[18] Alexandre C., Jacinto A., Ingham P.W. (1996). Transcriptional activation of hedgehog target genes in Drosophila is mediated directly by the cubitus interruptus protein, a member of the GLI family of zinc finger DNA-binding proteins. Genes Dev..

[19] Ruiz i Altaba A. (1998). Combinatorial Gli gene function in floor plate and neuronal inductions by Sonic hedgehog. Development.

[20] Cochrane C.R., Szczepny A., Watkins D.N., Cain J.E. (2015). Hedgehog Signaling in the Maintenance of Cancer Stem Cells. Cancers.

[21] Scales S., de Sauvage F. (2009). Mechanisms of Hedgehog pathway activation in cancer and implications for therapy. Trends Pharmacol. Sci..

[22] Hanna A., Shevde L.A. (2016). Hedgehog signaling: Modulation of cancer properies and tumor mircroenvironment. Mol. Cancer.

[23] Gorlin R.J. (1987). Nevoid basal-cell carcinoma syndrome. Medicine.

[24] Hahn H., Wicking C., Zaphiropoulous P.G., Gailani M.R., Shanley S., Chidambaram A., Vorechovsky I., Holmberg E., Unden A.B., Gillies S. (1996). Mutations of the human homolog of Drosophila patched in the nevoid basal cell carcinoma syndrome. Cell.

[25] Johnson R.L., Rothman A.L., Xie J., Goodrich L.V., Bare J.W., Bonifas J.M., Quinn A.G., Myers R.M., Cox D.R., Epstein E.H. (1996). Human homolog of patched, a candidate gene for the basal cell nevus syndrome. Science.

[26] Svärd J., Rozell B., Toftgård R., Teglund S. (2009). Tumor suppressor gene co-operativity in compound Patched1 and suppressor of fused heterozygous mutant mice. Mol. Carcinog..

[27] Theunissen J.W., De Sauvage F.J. (2009). Paracrine Hedgehog Signaling in Cancer. Cancer Res..

[28] Yauch R.L., Gould S.E., Scales S.J., Tang T., Tian H., Ahn C.P., Marshall D., Fu L., Januario T., Kallop D. (2008). A paracrine requirement for hedgehog signalling in cancer. Nature.

[29] Nolan-Stevaux O., Lau J., Truitt M.L., Chu G.C., Hebrok M., Fernández-Zapico M.E., Hanahan D. (2009). GLI1 is regulated through Smoothened-independent mechanisms in neoplastic pancreatic ducts and mediates PDAC cell survival and transformation. Genes Dev..

[30] Tian H., Callahan C.A., Dupree K.J., Darbonne W.C., Ahn C.P., Scales S.J., de Sauvage F.J. (2009). Hedgehog signaling is restricted to the stromal compartment during pancreatic carcinogenesis. Proc. Natl. Acad. Sci. USA.

[31] Lindemann R.K. (2008). Stroma-initiated Hedgehog signaling takes center stage in B-cell lymphoma. Cancer Res..

[32] Dierks C., Grbic J., Zirlik K., Beigi R., Englund N.P., Guo G.R., Veelken H., Engelhardt M., Mertelsmann R., Kelleher J.F. (2007). Essential role of stromally induced hedgehog signaling in B-cell malignancies. Nat. Med..

[33] Peacock C.D., Wang Q., Gesell G.S., Corcoran-Schwartz I.M., Jones E., Kim J., Devereux W.L., Rhodes J.T., Huff C.A., Beachy P.A. (2007). Hedgehog signaling maintains a tumor stem cell compartment in multiple myeloma. Proc. Natl. Acad. Sci. USA.

[34] Chen K., Huang Y.H., Chen J.L. (2013). Understanding and targeting cancer stem cells: Therapeutic implications and challenges. Acta Pharmacol. Sin..

[35] Gu D., Xie J. (2015). Non-Canonical Hh Signaling in Cancer-Current Understanding and Future Directions. Cancers.

[36] Brennan D., Chen X., Cheng L., Mahoney M., Riobo N.A. (2012). Noncanonical Hedgehog signaling. Vitam. Horm..

[37] Blotta S., Jakubikova J., Calimeri T., Roccaro A.M., Amodio N., Azab A.K., Foresta U., Mitsiades C.S., Rossi M., Todoerti K. (2012). Canonical and noncanonical Hedgehog pathway in the pathogenesis of multiple myeloma. Blood.

[38] Jeng K.S., Sheen I.S., Jeng W.J., Yu M.C., Hsiau H., Chang F.Y. (2013). High expression of Sonic Hedgehog signaling pathway genes indicates a risk of recurrence of breast carcinoma. Onco-Targets Ther..

[39] Zhao C., Chen A., Jamieson C.H., Fereshteh M., Abrahamsson A., Blum J., Kwon H.Y., Kim J., Chute J.P., Rizzieri D. (2009). Hedgehog signalling is essential for maintenance of cancer stem cells in myeloid leukaemia. Nature.

[40] Uhlén M., Björling E., Agaton C., Szigyarto C.A., Amini B., Andersen E., Andersson A.C., Angelidou P., Asplund A., Asplund C. (2005). A human protein atlas for normal and cancer tissues based on antibody proteomics. Mol. Cell. Proteomics.

[41] Im S., Choi H.J., Yoo C., Jung J.H., Jeon Y.W., Suh Y.J., Kang C.S. (2013). Hedgehog related protein expression in breast cancer: Gli-2 is associated with poor overall survival. Korean J. Pathol..

[42] Riaz N., Idress R., Habib S., Azam I., Lalani E.M. (2018). Expression of Androgen Receptor and Cancer Stem Cell Markers (CD44(+)/CD24(−) and ALDH1(+)): Prognostic Implications in Invasive Breast Cancer. Transl. Oncol..

[43] Papadopoulos V., Tsapakidis K., Riobo Del Galdo N.A., Papandreou C.N., Del Galdo F., Anthoney A., Sakellaridis N., Dimas K., Kamposioras K. (2016). The Prognostic Significance of the Hedgehog Signaling Pathway in Colorectal Cancer. Clin. Colorectal Cancer.

[44] Hasanovic A., Ruggiero C., Jung S., Rapa I., Signetti L., Ben Hadj M., Terzolo M., Beuschlein F., Volante M., Hantel C. (2018). Targeting the multidrug transporter Patched potentiates chemotherapy efficiency on adrenocortical carcinoma in vitro and in vivo. Int. J. Cancer.

[45] Saze Z., Terashima M., Kogure M., Ohsuka F., Suzuki H., Gotoh M. (2012). Activation of the sonic hedgehog pathway and its prognostic impact in patients with gastric cancer. Dig. Surg..

[46] Xu X., Ding H., Rao G., Arora S., Saclarides C.P., Esparaz J., Gattuso P., Solorzano C.C., Prinz R.A. (2012). Activation of the Sonic Hedgehog pathway in thyroid neoplasms and its potential role in tumor cell proliferation. Endocr. Relat. Cancer.

[47] Zhu W., You Z., Li T., Yu C., Tao G., Hu M., Chen X. (2011). Correlation of hedgehog signal activation with chemoradiotherapy sensitivity and survival in esophageal squamous cell carcinomas. Jpn. J. Clin. Oncol..

[48] Gonnissen A., Isebaert S., Perneel C., McKee C.M., Van Utterbeeck F., Lerut E., Verrill C., Bryant R.J., Joniau S., Muschel R.J. (2018). Patched 1 Expression Correlates with Biochemical Relapse in High-Risk Prostate Cancer Patients. Am. J. Pathol..

[49] Taipale J., Cooper M., Maiti T., Beachy P. (2002). Patched acts catalytically to suppress the activity of Smoothened. Nature.

[50] Low J.A., de Sauvage F.J. (2010). Clinical experience with Hedgehog pathway inhibitors. J. Clin. Oncol..

[51] Bijlsma M.F., Spek A., Zivkovic D., van de Water S., Rezaee F., Peppelenbosch M.P. (2006). Repression of smoothened by patched-dependent (pro-)vitamin D3 secretion. PLoS Biol..

[52] Rohatgi R., Milenkovic L., Scott M.P. (2007). Patched1 regulates hedgehog signaling at the primary cilium. Science.

[53] Corcoran R., Scott M. (2006). Oxysterols stimulate Sonic hedgehog signal transduction and proliferation of medulloblastoma cells. Proc. Natl. Acad. Sci. USA.

[54] Dwyer J.R., Sever N., Carlson M., Nelson S.F., Beachy P.A., Parhami F. (2007). Oxysterols are novel activators of the hedgehog signaling pathway in pluripotent mesenchymal cells. J. Biol. Chem..

[55] Yavari A., Nagaraj R., Owusu-Ansah E., Folick A., Ngo K., Hillman T., Call G., Rohatgi R., Scott M.P., Banerjee U. (2010). Role of lipid metabolism in smoothened derepression in hedgehog signaling. Dev. Cell.

[56] Cooper M.K., Wassif C.A., Krakowiak P.A., Taipale J., Gong R., Kelley R.I., Porter F.D., Beachy P.A. (2003). A defective response to Hedgehog signaling in disorders of cholesterol biosynthesis. Nat. Genet..

[57] Altmann S.W., Davis H.R., Zhu L.J., Yao X., Hoos L.M., Tetzloff G., Iyer S.P., Maguire M., Golovko A., Zeng M. (2004). Niemann-Pick C1 Like 1 protein is critical for intestinal cholesterol absorption. Science.

[58] Davis H.R., Zhu L.J., Hoos L.M., Tetzloff G., Maguire M., Liu J., Yao X., Iyer S.P., Lam M.H., Lund E.G. (2004). Niemann-Pick C1 Like 1 (NPC1L1) is the intestinal phytosterol and cholesterol transporter and a key modulator of whole-body cholesterol homeostasis. J. Biol. Chem..

[59] Infante R.E., Abi-Mosleh L., Radhakrishnan A., Dale J.D., Brown M.S., Goldstein J.L. (2008). Purified NPC1 protein. I. Binding of cholesterol and oxysterols to a 1278-amino acid membrane protein. J. Biol. Chem..

[60] Liu R., Lu P., Chu J., Sharom F. (2009). Characterization of fluorescent sterol binding to purified human NPC1. J. Biol. Chem..

[61] Bidet M., Joubert O., Lacombe B., Ciantar M., Nehmé R., Mollat P., Brétillon L., Faure H., Bittman R., Ruat M., Mus-Veteau I. (2011). The hedgehog receptor patched is involved in cholesterol transport. PLoS ONE.

[62] Kuwabara P., Labouesse M. (2002). The sterol-sensing domain: Multiple families, a unique role?. Trends Genet..

[63] Martin V., Carrillo G., Torroja C., Guerrero I. (2001). The sterol-sensing domain of Patched protein seems to control Smoothened activity through Patched vesicular trafficking. Curr. Biol..

[64] Strutt H., Thomas C., Nakano Y., Stark D., Neave B., Taylor A.M., Ingham P.W. (2001). Mutations in the sterol-sensing domain of Patched suggest a role for vesicular trafficking in Smoothened regulation. Curr. Biol..

[65] Khaliullina H., Panáková D., Eugster C., Riedel F., Carvalho M., Eaton S. (2009). Patched regulates Smoothened trafficking using lipoprotein-derived lipids. Development.

[66] Haas D., Muenke M. (2010). Abnormal sterol metabolism in holoprosencephaly. Am. J. Med. Genet. C Semin. Med. Genet..

[67] Minogue S., Chu K.M., Westover E.J., Covey D.F., Hsuan J.J., Waugh M.G. (2010). Relationship between phosphatidylinositol 4-phosphate synthesis, membrane organization, and lateral diffusion of PI4KIIalpha at the trans-Golgi network. J. Lipid Res..

[68] Lindström E., Shimokawa T., Toftgård R., Zaphiropoulos P.G. (2006). PTCH mutations: Distribution and analyses. Hum. Mutat..

[69] Soloviev A., Gallagher J., Marnef A., Kuwabara P.E. (2011). *C. elegans* patched-3 is an essential gene implicated in osmoregulation and requiring an intact permease transporter domain. Dev. Biol..

[70] Davies J.P., Levy B., Ioannou Y.A. (2000). Evidence for a Niemann-pick C (NPC) gene family: Identification and characterization of NPC1L1. Genomics.

[71] Guan L., Nakae T. (2001). Identification of essential charged residues in transmembrane segments of the multidrug transporter MexB of Pseudomonas aeruginosa. J. Bacteriol..

[72] Bidet M., Tomico A., Martin P., Guizouarn H., Mollat P., Mus-Veteau I. (2012). The Hedgehog receptor patched functions in multidrug transport and chemotherapy resistance. Mol. Cancer Res..

[73] Detmer K., Walker A.N., Jenkins T.M., Steele T.A., Dannawi H. (2000). Erythroid differentiation in vitro is blocked by cyclopamine, an inhibitor of hedgehog signaling. Blood Cells Mol. Dis..

[74] Hammer G.D., Fassnacht M., Lalli E. (2011). Adrenal cancer: Scientific advances. Mol. Cell. Endocrinol..

[75] Else T., Kim A.C., Sabolch A., Raymond V.M., Kandathil A., Caoili E.M., Jolly S., Miller B.S., Giordano T.J., Hammer G.D. (2014). Adrenocortical carcinoma. Endocr. Rev..

[76] Vander Heiden M.G., Cantley L.C., Thompson C.B. (2009). Understanding the Warburg effect: The metabolic requirements of cell proliferation. Science.

[77] Damaghi M., Wojtkowiak J.W., Gillies R.J. (2013). pH sensing and regulation in cancer. Front. Physiol..

[78] Kato Y., Ozawa S., Miyamoto C., Maehata Y., Suzuki A., Maeda T., Baba Y. (2013). Acidic extracellular microenvironment and cancer. Cancer Cell Int..

[79] Gerweck L.E., Seetharaman K. (1996). Cellular pH gradient in tumor versus normal tissue: Potential exploitation for the treatment of cancer. Cancer Res..

[80] Fiorini L., Mus-Veteau I. (2016). Method to Screen Multidrug Transport Inhibitors Using Yeast Overexpressing a Human MDR Transporter. Methods Mol. Biol..

[81] Fiorini L., Tribalat M.A., Sauvard L., Cazareth J., Lalli E., Broutin I., Thomas O.P., Mus-Veteau I. (2015). Natural paniceins from mediterranean sponge inhibit the multidrug resistance activity of Patched and increase chemotherapy efficiency on melanoma cells. Oncotarget.

[82] Monachon M.A., Burkard W.P., Jalfre M., Haefely W. (1972). Blockade of central 5-hydroxytryptamine receptors by methiothepin. Naunyn. Schmiedeberg Arch. Pharmacol..

[83] Dall’Olio R., Vaccheri A., Montanaro N. (1985). Reduced head-twitch response to quipazine of rats previously treated with methiothepin: Possible involvement of dopaminergic system. Pharmacol. Biochem. Behav..

[84] Sridhar R., Dwivedi C., Anderson J., Baker P.B., Sharma H.M., Desai P., Engineer F.N. (1992). Effects of verapamil on the acute toxicity of doxorubicin in vivo. J. Natl. Cancer Inst..

[85] Colombo T., Zucchetti M., D’Incalci M. (1994). Cyclosporin A markedly changes the distribution of doxorubicin in mice and rats. J. Pharmacol. Exp. Ther..

[86] Bellamy W.T., Peng Y.M., Odeleye A., Ellsworth L., Xu M.J., Grogan T.M., Weinstein R.S. (1995). Cardiotoxicity in the SCID mouse following administration of doxorubicin and cyclosporin A. Anticancer Drugs.

[87] Magdy T., Burmeister B.T., Burridge P.W. (2016). Validating the pharmacogenomics of chemotherapy-induced cardiotoxicity: What is missing?. Pharmacol. Ther..

